# A Computer-Based Automated Algorithm for Assessing Acinar Cell Loss after Experimental Pancreatitis

**DOI:** 10.1371/journal.pone.0110220

**Published:** 2014-10-24

**Authors:** John F. Eisses, Amy W. Davis, Akif Burak Tosun, Zachary R. Dionise, Cheng Chen, John A. Ozolek, Gustavo K. Rohde, Sohail Z. Husain

**Affiliations:** 1 Pediatrics, University of Pittsburgh, Children's Hospital of Pittsburgh of UPMC, Pittsburgh, Pennsylvania, United States of America; 2 Pathology, University of Pittsburgh, Children's Hospital of Pittsburgh of UPMC, Pittsburgh, Pennsylvania, United States of America; 3 Biomedical and Electrical and Computer Engineering, Center for Bioimage Informatics, Carnegie Mellon University, Pittsburgh, Pennsylvania, United States of America; University of Szeged, Hungary

## Abstract

The change in exocrine mass is an important parameter to follow in experimental models of pancreatic injury and regeneration. However, at present, the quantitative assessment of exocrine content by histology is tedious and operator-dependent, requiring manual assessment of acinar area on serial pancreatic sections. In this study, we utilized a novel computer-generated learning algorithm to construct an accurate and rapid method of quantifying acinar content. The algorithm works by learning differences in pixel characteristics from input examples provided by human experts. HE-stained pancreatic sections were obtained in mice recovering from a 2-day, hourly caerulein hyperstimulation model of experimental pancreatitis. For training data, a pathologist carefully outlined discrete regions of acinar and non-acinar tissue in 21 sections at various stages of pancreatic injury and recovery (termed the “ground truth”). After the expert defined the ground truth, the computer was able to develop a prediction rule that was then applied to a unique set of high-resolution images in order to validate the process. For baseline, non-injured pancreatic sections, the software demonstrated close agreement with the ground truth in identifying baseline acinar tissue area with only a difference of 1%±0.05% (p = 0.21). Within regions of injured tissue, the software reported a difference of 2.5%±0.04% in acinar area compared with the pathologist (p = 0.47). Surprisingly, on detailed morphological examination, the discrepancy was primarily because the software outlined acini and excluded inter-acinar and luminal white space with greater precision. The findings suggest that the software will be of great potential benefit to both clinicians and researchers in quantifying pancreatic acinar cell flux in the injured and recovering pancreas.

## Introduction

Acute pancreatitis is marked by acinar cell injury that leads to a transient loss of acinar tissue [1], [2], [3]. In the initial phase of pancreatitis, the amount of acinar dropout is a useful index to assess the extent of injury. Once acinar cell loss has reached a nadir, its reconstitution can be used to gauge the kinetics of regeneration and recovery of the pancreas [4], [5]. Acinar cells comprise the majority of the parenchyma and are a major contributor to the renewal of exocrine tissue [6]. Histological analysis of sectioned pancreatic tissue remains the gold standard for identifying and quantifying the extent of acinar loss [7], [8]. Historically, evaluation of acinar cell content has been qualitatively measured through the application of grading schemes, which provide, at best, a semi-quantitative estimate. Hematoxylin and eosin (HE) stained sections are evaluated by a trained individual for acinar cell morphology and, typically, multiple fields of view are averaged. This type of quantification is laborious and time-consuming because it requires a microscopic examination of multiple tissue sections from the entire sectioned pancreas. For this reason, most papers provide representative images that only show trends in acinar content.

Recently, computer driven segmentation algorithms have arisen to help automate the analysis of digital images [9], [10], [11]. Several commercial applications are available (i.e. MetaMorph (Molecular Devices, LLC,Sunnyvale, CA) or Volocity (Perkin-Elmer, Waltham, MA), but they are limited to a few parameters such as pixel number or intensity and typically require fluorescent immunostaining of regions of interest (ROI) [12], [13]. By contrast, a recently developed computer-learning algorithm utilizes a unique system of biomedical image segmentation [14], by identifying pixels and classifying them based on surrounding pixel neighborhoods. The technique can be applied to multiple imaging modalities including fluorescent microscopy, brightfield microscopy, and cross-sectional imaging. Using manually segmented data provided by a human expert as training data, the computer learns to identify structures by analyzing the neighboring pixels. A prediction rule is generated that is subsequently used in an automated fashion. The benefits of this type of image segmentation strategy are improved accuracy and speed, and the generation of quantitative results.

In this study, we employed an automated computer-learning system that employs a newly developed image segmentation algorithm to accurately and rapidly quantify pancreatic acini from fixed HE stained pancreatic sections following injury from an experimental pancreatitis model in mice. The computer accurately determined the percentage of tissue occupied by pancreatic acini from digital images of tissue sections at three distinct time points of pancreatic injury and recovery. Importantly, the computer was able to process the same number of images fifteen times faster and in some cases more precisely when compared to manual human segmentation.

## Materials and Methods

### Reagents and animals

Caerulein was purchased from Sigma-Aldrich (St. Louis, MO). Six-week-old male Swiss Webster mice (Charles River, Wilmington, MA) weighing 20–25 grams were fed standard laboratory chow with free access to water. All animal experiments were performed using a protocol approved by the University of Pittsburgh Institutional Animal Care and Use Committee.

Pancreatic injury and acinar dropout in mice were induced by a standard pancreatitis induction method using the cholecystokinin (CCK) analog caerulein at a hyper-stimulatory dose of 50 µg/kg. Mice received 8 hourly intra-peritoneal caerulein injections on 2 consecutive days (days −1 and 0). Groups of mice were euthanized by CO_2_ asphyxiation on days 1, 3, and 7 after the last day of caerulein injections. The pancreas was removed, placed in 4% PFA, and then processed for HE staining.

### Histologic preparation and generation of digital images for analysis

Pancreatic sections were processed by a single facility (the Histology Core at the Children's Hospital of Pittsburgh) in order to standardize the HE staining process for each of the pancreatic samples. Stained sections were used to generate images using the automated biomedical image segmentation algorithm. The digital images were acquired with an Aperio whole slide scanner (Aperio ePathology Solutions, Leica Microsystems, Inc., Buffalo Grove, IL. USA) using a magnification of 400x, which yielded a file size of 750–900 megabytes. The spatial resolution of images from the scanner at this magnification was 0.14 µm/pixel. To facilitate image analysis, the larger image was parsed into smaller discrete images that were used for training and testing of the computer generated algorithm. The smaller images had spatial resolutions of 0.41 µm/pixel.

### Segmentation of digital images for analysis

**Figure 1:**
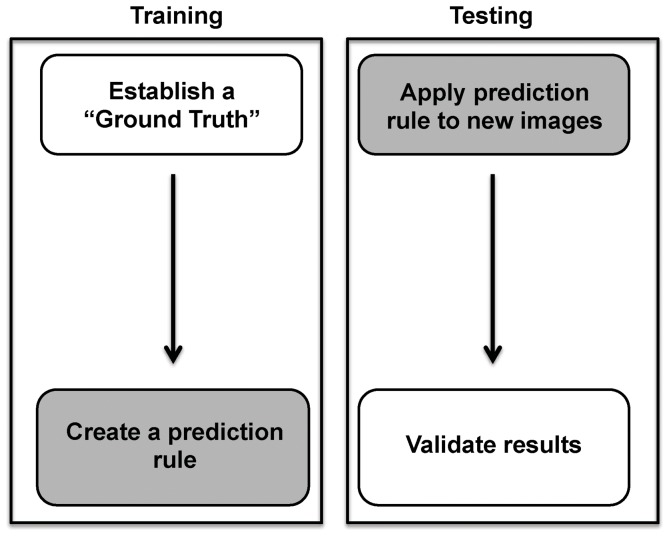
Flow diagram depicting the training and testing phases of the computer algorithm. A human expert establishes the “ground truth.” The computer then creates a prediction rule by modeling image characteristics and analyzing pixel neighborhoods. In the testing phase, the computer applies the prediction rule to a new set of images. The computer results are finally validated by the human expert. White and gray boxes refer to the human expert and computer actions, respectively.

**Figure 2:**
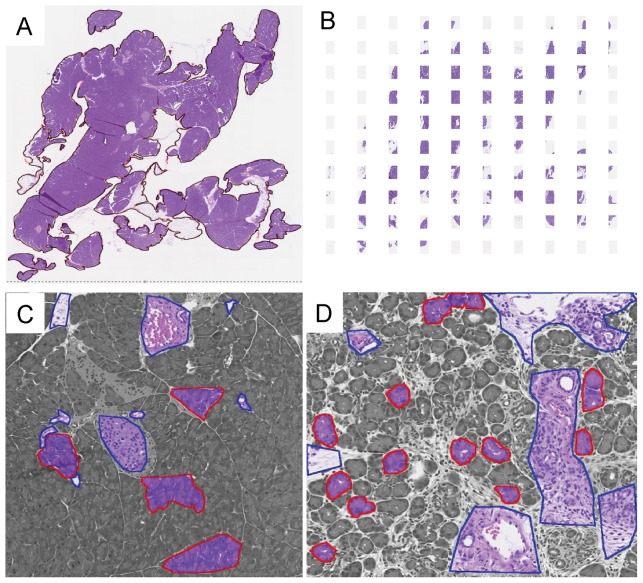
A human expert initiates the training phase of computer learning by identifying the “ground truth.” (A) HE staining of a whole pancreas slice. Regions to be included are outlined in black. (B) The whole image is parsed into smaller patches. (C, D) A pathologist then identifies acinar structures (in red) and excludes non-acini (in blue) from a heterogeneous array of pancreatic tissues in order to construct the ground truth.

The flowchart of the proposed automated image segmentation approach is depicted in Figure 1. There are two identifiable and distinct stages of the method. The first stage, the training stage, uses pathologist analyzed input data (termed the “ground truth”). The ground truth consists of a set of training images in which structures of interest (in this case, acini) are outlined by an expert pathologist. Specifically, 21 training images were chosen in order to adequately sample the variations in acinar appearance following pancreatic injury, as defined by a pathologist. In a 2-dimensional cross-section, acini are circular to oval shaped structures that are made up of acinar cells, whose apical portions line a centrally localized lumen. The acinar cells are columnar to pyramidal epithelial cells. The nuclei within acinar cells are located basolaterally, and their nucleolus is evident. The endoplasmic reticulum of acinar cells is abundant and stain intensely basophilic by HE. At the apical borders of acinar cells, zymogen granules are present and stain pink with eosin. The characteristic staining pattern by HE allows ready identification and discrimination from other cell types [15]. Using the caerulein hyperstimulation pancreatitis model, we derived the ground truth by having an expert pathologist identify acinar cells in pancreatic HE sections. During the training phase, the stained pancreas sections were scanned and stored as digitized images (Figure 2A). Using this large image, pancreas structures were outlined to identify a denominator (i.e. total pancreas area for each scanned slide). In the example provided in Figure 2, the pancreatic tissue included in the analysis was outlined in black, and white space was excluded from the analysis and area calculations (Figure 2A). The large image was then parsed into smaller images that could be easily handled by human operators (Figure 2B). The pathologist was asked to outline a few representative areas of acinar (in red) and non-acinar tissue (in blue) in these parsed images from baseline normal pancreas as well as injured and recovering tissue (Figure 2C&D).

The image regions containing the representative acinar and non-acinar tissue were then used to train a pattern recognition system to identify similar structures (areas) in any other image. A more comprehensive detail of the methods and concepts have been previously described [14], [16]. For completeness, however, we provide here the following description. The system utilizes labeled input pixel data to train a mathematical function that can be used to predict whether any similar region from another image would be labeled acinar or non-acinar by the same pathologists. Let 
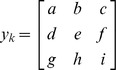
represent a 3×3 array of pixel intensities (encoded in the variables *a*, *b*, …) chosen from a particular region. For example, let *e* correspond to a pixel intensity that belongs to a region labeled as acinar by our trained pathologist. In this case, the intensities corresponding to *a* through *i* in the array are the pixel intensities in the immediate vicinity of *e*. Because the images are in color, in this case, *e* is not a single number but, in fact, a triad of numbers corresponding to the Red, Green, and Blue (RGB) components at that location. Our explanation will still apply, since ultimately we apply the algorithm being described here to each color independently and combined results. Now let *Y* =  *{y_1_, y_2_, …, y_k_, …, y_m_}* represent a set of *M* such vectors, each containing information from a pixel labeled as acinar. Each vector *y_k_* is then processed to remove any effects related to rotation. Therefore, we simply reorder each element in each *y_k_* so that they are organized as a 9×1 vector of decreasing intensities. Similarly, *X*  =  *{x_1_, x_2_, …, x_k_, …, x_n_}* represent a set of vectors (each vector is organized in order of decreasing intensity). Note that the number of representative pixels (*M* for set *Y*, and *N* for set *X*) need not be the same. We utilize a nearest neighbor approach to assign a label (acinar vs. non-acinar) for any array containing pixel intensities from a particular unknown pixel z*_j_*. That is, let 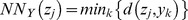 and 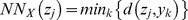 with *d*(.,.) corresponding to the standard Euclidean distance between two vectors. The class assignment for pixel z*_j_* is given by testing which of the two quantities 
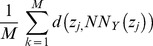
(1)or 
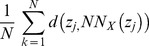
(2)is bigger.

The above was a brief summary of the nearest neighbor approach we have used in order to automatically highlight and separate acinar from non-acinar regions in a given whole slide image. The approach is described succinctly in order to avoid an overly elaborate method presentation, although further details of the method have been recently published [14], [16]. In particular, Chen et al (2013) describe a method to select the “most important” pixels in the training set *X*, and *Y* described above, so that the computation can be achieved in a reasonable time. This is done because with a few hours of work in a modern workstation, a pathologist is able to select pixel training sets from whole slide images which contain several millions of pixels. If the sums in equations (1) and (2) have to sum over many millions of pixels for classifying a single given input pixel, the amount of time needed for segmenting a given whole slide image would be excessive. In addition, we have used a multi-scale strategy which is previously described [14]. Briefly, the approach highlighted above is repeated using 3×3 intensity neighborhoods chosen from different scales (different scales of a whole slide images can be simulated digitally using low pass filters). This is used to ensure that local information at different levels is included for detecting acinar regions. Once a whole slide image has been segmented, the computer's result is then compared to a parallel analysis performed by an expert in order to validate the prediction rule.

The segmentation by the human expert was accomplished by outlining acini in Adobe Photoshop (Adobe Systems Inc., San Jose, Ca. USA) using a 19-inch digital Bosto Kingtee 19MA interactive graphics monitor (Bosto International Co. LTD, Shenzhen, China). Non-acinar tissue including islets of Langerhans, connective tissue between lobules, large ducts, and larger blood vessels were excluded.

Subsequently, the computer “learned” the image characteristics of the outlined areas by generating a set of classifiers that were specific for acinar tissue. In the training stage, the computer normalized the input data and modeled scaling and orientations of the input data via filtering and resampling. Then it extracted 3×3 pixel neighborhood intensities for each pixel in color and repeated this procedure for 3 different resolutions of the original image, which in addition to capturing local information about a particular pixel also gathers spatial information about that pixel's neighborhood. After extracting features for each pixel, the algorithm then selected a portion of the information via a modified data selection method that utilizes neighborhood voting to make the data selection process more accurate and efficient [16]. The system trained several classifiers at different image resolutions.

In the next distinct phase, called the testing phase, the computer automated prediction rule was applied on new images at different image resolutions. There were 6 images for baseline and 12 images for injured pancreas used during this phase. Segmentations on new images were obtained by classifying every single pixel using 1-NN (nearest neighbor) classifier, followed by majority voting (over different image resolutions) spatially within local windows [14]. The computers analysis of acinar area for the test images was then compared to two human experts (i.e. a board-certified pathologist [A.W.D.] and an experienced investigator in pancreatic injury [J.F.E.]) analysis to validate the computer's result. Acinar content was determined by counting pixels classified as acinar after images were segmented whether performed by the computer or by a human expert. The acinar content was then expressed as a percentage of the whole pancreas tissue for each image.

Finally, the prediction rule was applied to images taken from sections of pancreatic tissue at discrete time points after caerulein hyperstimulation. There were 3 images from baseline, 6 images from Day 1, 6 images from Day 3, and 6 images from Day 7 used for analysis. The computer's analysis was compared to two human experts to determine accuracy of the computer.

### Statistical analysis

Accuracy was calculated using the following formula:
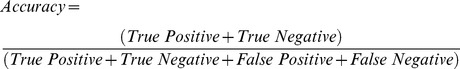



True positives represent acini manually segmented by the human expert as well as the computer. True negatives are non-acinar tissue correctly excluded by both human expert and computer. False positives are non-acinar tissue identified by the computer but not the human expert. False negatives are acini that are excluded by the computer but included by the human expert.

In addition, the computer-generated prediction rule was assessed using the F_1_ score, which is a measure of the performance of the computer-derived set of classifiers, or the prediction rule. The F_1_ score assesses both the precision and recall of the computer's prediction rule. Precision is defined as the number of correct results divided by the number of all returned results, and recall is defined as the number of correct results divided by the number of results that should have been returned. The F_1_ score is a weighted average of both precision and recall, in which 1 is the best and 0 the worst score. Results were expressed as mean ± standard deviation, unless otherwise stated. Statistical significance between groups was determined using a Student's t-test and defined as a p value ≤0.05.

### Software

For software access and information please visit: http://tango.andrew.cmu.edu/~gustavor/AcinarAnalysisFramework.zip and http://www.andrew.cmu.edu/user/gustavor/software.html.

## Results

**Figure 3:**
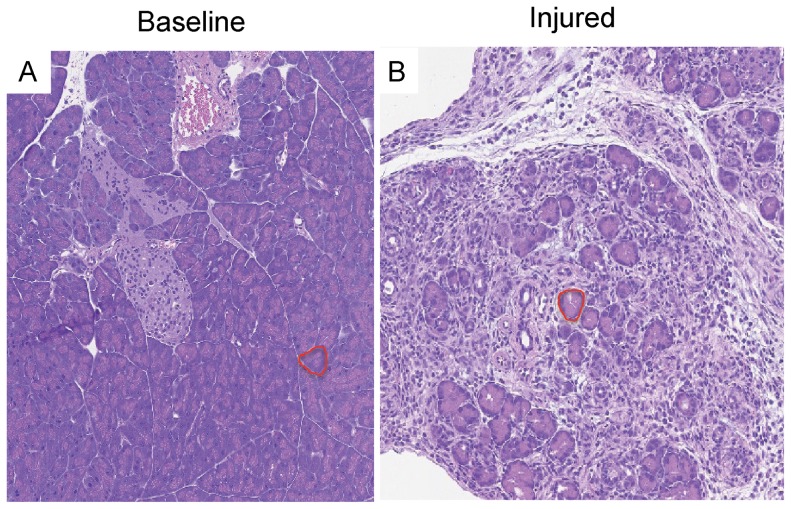
Caerulein hyperstimulation causes marked acinar cell loss. Representative pancreas sections stained with HE isolated from mice (A) at baseline (uninjured) or (B) 3 days after caerulein hyperstimulation pancreatitis. For demonstration, a single acinus in each panel is outlined in red.

The predominant cell type in the pancreas is the acinar cell and the predominant microarchitectural structure is the acinus, which is estimated to make up 80-90% of the parenchyma (Figure 3A) [17], [18]. In contrast, severe acinar cell loss is observed within 3 days of stimulation with the CCK analogue, caerulein (Figure 3B). In this highly reproducible model of experimental pancreatitis, several processes impact acinar cell integrity [19]. They include features of the acinar cell such as the premature intra-acinar activation of proteases [19], [20], [21], as well as extra-acinar sequalae. Damage signals from the pancreas, starting with the acinar cell [22], initiate a robust inflammatory infiltration into the pancreatic parenchyma with accompanying stromal edema and individual acinar cell swelling [23], [24]. In this new inflammatory milieu and with larger-sized acini, the computer-learning algorithm must develop a prediction rule that can still accurately quantify acini containing these variations.

**Figure 4:**
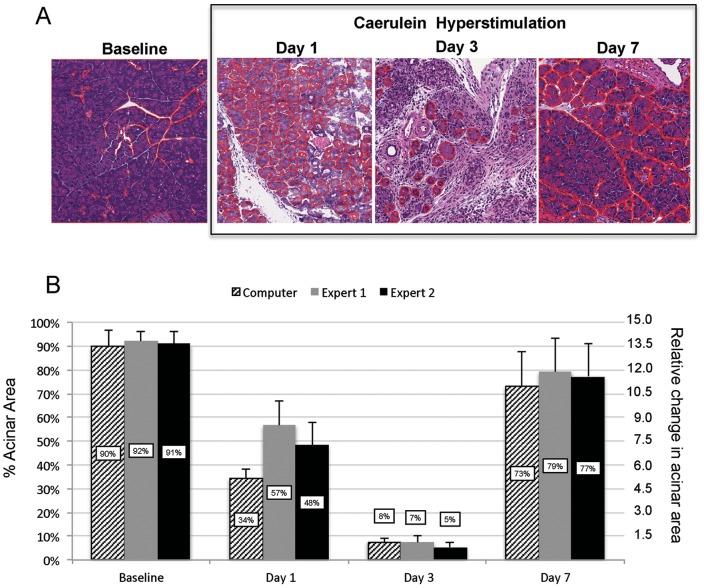
The automated computer-learning system can be used to track acinar cell loss and recovery after acute pancreatic injury. (A) Representative HE pancreas sections at baseline and from several time points after caerulein hyperstimulation, with acinar structures outlined in red. (B) Quantification of acinar area generated by the computer algorithm and two human experts. (n = 6 patches for each time point). The graph shows absolute percent acinar area compared to total pancreatic tissue (left axis) as well as the relative change in acinar tissue as normalized by Day 3 acinar content (right axis). Overall sensitivity  = 88%, specificity  = 86%, accuracy  = 93%, and F_1_ score  = 0.86.

The computer algorithm must accommodate subtle changes in acinar shape, color, and cellular composition in order to accurately identify individual acinar cells irrespective of the degree of injury to each acinar cell. To determine if the algorithm was capable of detecting these subtle differences in the exocrine pancreas, initial analysis of two extreme conditions (non-injured and injured) were used. To achieve this goal, two human experts independently determined acinar content in baseline and injured pancreas samples and then their results were compared to the computer's results (Figure 4, Baseline and Day 3). Each of the three results for acinar content between the computer and the two pathologists was similar. Overall, the computer-based prediction rule had a sensitivity of 88%, specificity of 86%, and accuracy of 93% as determined for all images segmented. In digital images of baseline (uninjured) pancreas tissue, acinar content was 90%±7%, 92%±4%, and 91%±5%, for the computer, expert 1, and expert 2, respectively (p = 0.19). In digital images of maximally injured pancreas tissue (Day 3), it was 8%±2%, 7%±3%, and 5%±2%, respectively (p = 0.47). The overall F_1_ score for the computer segmentation algorithm was 0.86, which demonstrates strong correlation with the human experts. These data indicate that the machine-learning algorithm accurately identifies acinar content in both baseline and injured pancreatic tissue. Importantly, the computer was approximately fifteen times faster than the human analyst. For example, the computer completed analysis of 42 images consisting of both baseline and injured pancreatic sections in two hours, while the pathologists each took over 30 hours to complete the same analysis.

**Figure 5:**
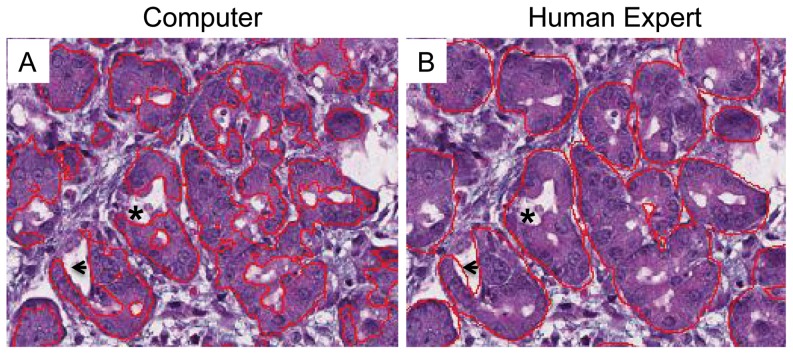
The computer-learning system outlines acini more precisely than can be performed manually. In the representative HE pancreatic sections from Day 1 after caerulein hyperstimulation, (A) the computer outlines acini (in red) more tightly (arrowheads) and is more likely to exclude intra-acini white space (asterisks) than can be performed manually by (B) human experts.

During pancreatitis, there is a continuum of injury and regeneration that ultimately restores the pancreatic parenchyma to a recovered state. The exocrine pancreas demonstrates marked changes in morphology and volume from subtle changes early in the injury process, followed by extreme acinar loss and transient replacement with non-acinar structures. However, the acinar tissue almost completely regenerates. Following acinar content during this dynamic process of injury and recovery is helpful in determining the timing and degree of recovery following an acute pancreatic insult. Following caerulein hyperstimulation, there is marked acinar cell loss early, followed by profound exocrine regeneration by Day 7 (i.e. 7 days after the last caerulein injection) [5], [25]. We used the computer-learning prediction rule to quantify acinar dropout and recovery over this time course of dropout and regeneration. Whereas the previous determination of acinar area from baseline and maximally injured tissue was used to validate the accuracy of the prediction rule, the established prediction rule was hence applied to newly derived images from discrete time points after caerulein injury. A dramatic loss of acinar content was noted from baseline (90%±7% of total parenchymal area) to Day 3 (8%±2%), and a reconstitution of acinar content was observed by Day 7 (73%±14%) (Figure 4). To further cross-validate the images analyzed by the computer, the two experts determined acinar area for the same images scored by the computer. There was concordance between the three reviewers (i.e. computer; expert 1; expert 2) at baseline (90%±7%; 92%±4%; 91%±5%), on Day 3 (8%±2%; 7%±3%; 6%±2%), and on Day 7 (73%±14%; 79%±14%; 77±14%). However, for Day 1 images, at a time when injured acini are swollen and deformed, there was less agreement among the three. There was a 23% difference between the computer and expert 1 (p = 0.0002) and a 14% difference between the computer and expert 2 (p = 0.003). Importantly, even the two human experts lacked close agreement on the amount of acinar content in the stained sections at Day 1. Whereas there was only an inter-observer variability of 2%±1% between the two experts at baseline or on Day 3 or Day 7, the difference at Day 1 was 9%±5% (p = 0.009). Upon closer analysis of the results from the computer segmentation for Day 1 images, we observed that the computer outlined the acinar borders and excluded white space (representing intralobular stroma, edema, and acinar lumens) more precisely compared to the human experts (Figure 5). This could account for the lower acinar content outputted by the computer for the Day 1 samples. In addition, due to the severe disruption of the microarchitecture and damage to acinar cells, in particular, disparity over what constituted a true acinar structure factored into inter-observer variability between both human experts as well as the computer compared to human experts in evaluation of Day 1 images. However, a potential limitation of the algorithm is that the computer also excluded larger cytoplasmic vacuoles within acinar cells. Overall, the computer generated prediction rule was able to reliably identify acini and subsequently calculate the percentage of acinar tissue from images in uninjured, injured, and recovering pancreas tissue following experimental pancreatitis.

## Discussion

We have employed an automated computer-learning system that accurately and rapidly quantifies pancreatic acinar area in a pancreatic injury model. To our knowledge, the current study is also the first to demonstrate use of a computer-learning algorithm to solve a pancreas-related problem. The method is novel in its approach compared to existing commercial software because in the machine-learning system, the computer creates its own identifiers in the form of a prediction rule rather than simply using a few code-defined characteristics [26], [27], [28]. This unique advantage allows the system to develop multiple color and pixel identifiers from digital images of HE stained pancreatic sections of uninjured and injured tissue. Similar technology has recently been used in several other pathology applications, including the identification of tumor cell nuclei in breast and prostate cancer [10], [29], [30] and apoptosis in cell lines [31].

The successful implementation of this automated machine-learning strategy relies exclusively on the quality of the training data provided by human operators. Evidence for this finding is that the computer's identification of acini and percentage calculation of acinar area was in close agreement with the two human experts in all cases where the human experts demonstrated low inter-observer variability. Thus, operators should have a high level of expertise in identifying the structures of interest before having the computer run a prediction rule.

Possibly the most powerful aspect of the machine-learning method is its ability to identify acini and calculate acinar area in a rapid fashion. Even the most seasoned human expert requires a large amount of time to manually outline acini and calculate acinar area from multiple whole slide images. Because of the high estimated time expenditure and, importantly, operator fatigue, most papers concerning pancreatic regeneration either report acinar content from only a few representative sections or provide a subjective, qualitative interpretation [32], [33], [34]. The subjective interpretation by a blinded human expert, however, does offer some advantages over a computer application. The expert considers additional parameters such as necrosis, edema, and inflammatory infiltration. Nonetheless, the computer can be trained to identify these aspects of pancreatic injury as well, a task that constitutes future studies. The advantage of the computer, in this case, is that once trained it can identify these structures more rapidly than a human expert, and the program can be used on any fixed HE stained pancreas slide, despite the mode of injury. Once trained to identify a specific injury parameter, there is no need to retrain the program for future experiments as long as HE staining is consistent. Other methods to quantify acinar tissue content are available, such as quantifying amylase or trypsinogen by western blot or immunofluorescence. For the latter, one can use commercial software (e.g. MetaMorph or Volocity) as well as open access programs such as ImageJ. Although reliable, these methods can be time consuming and costly. In addition, IHC staining can produce non-specific staining, particularly when staining for cytoplasmic enzymes that can leak into extracellular space following cellular injury. The computer algorithm, on the other hand, uses HE stained slides that are widely available, highly standardized and routinely archived. We believe that the computer-learning algorithm will be of great benefit to the pancreas field because it will make accurate quantification of pancreas tissue an expedited process. For instance, highly accurate estimates of quantity (as measured by area from a digital image) of exocrine pancreas can be readily done for an entirely sectioned pancreas. This accurate quantification would provide a precise gauge with which to determine subtle differences in regeneration models and therapeutic interventions. Also, a non-trivial advantage is that what took 30 hours on average for each human expert to outline, took only 2 hours for the computer to generate.

Although the supervised learning-based method for acinar detection from whole slide histopathology images we described in this study will greatly facilitate the quantitative analysis of large numbers of images. There is currently a technical limitation in widely using the method. The software was custom built at Carnegie Mellon University, and was written in the Matlab (Mathworks, Inc.) programming language. While certain software alternatives exist (e.g. ImageJ) that are freely available to the scientific community and are easy enough for non-expert users, the methods described above involve extensive computations that would be challenging for non-experts who only have access to a standard workstation. The computations were performed on a Beowulf-type computer cluster that utilized, in parallel, multiple processors. On average, if only one processor was used to segment a whole slide image, the computation time would take roughly 60 hours. Similarly, the training step (i.e. mining through the input-labeled data to determine classification rules) would take approximately 12 hours. Thus, at the present, these techniques, though accurate, require more extensive computational resources than typical programs that can be implemented in user-friendly packages. Future work, however, in this area is being devoted to reduce the computation time using more sophisticated mathematical algorithms, as well as to implement the programs in a free open source computer programming language that can be released free of charge to the academic community.

In summary, the computer-learning algorithm in this study provides accurate acinar cell identification and rapid assessment of acinar area from HE sections of the pancreas. We believe the automated machine-learning algorithm will be a valuable asset in quantifying pancreatic acinar cell content in pancreatic injury and regeneration models. The system is adaptable and can potentially, in future work, be used to accurately and rapidly assess multiple other pancreatic phenotypes of interest.
